# Reductio ad bacterium: the ubiquity of Bayesian “brains” and the goals of cognitive science

**DOI:** 10.3389/fpsyg.2012.00498

**Published:** 2012-11-15

**Authors:** Ben Sheredos

**Affiliations:** Ph.D. Program, Department of Philosophy, UC San DiegoLa Jolla, CA, USA

**A commentary on**

**Whatever next? Predictive brains, situated agents, and the future of cognitive science**

by Clark, A. (in press). Behav. Brain Sci.

If cognition is Clarkian, the class of extant cognizers includes nearly all living organisms. All organisms displaying circadian rhythmicity meet Clark's criteria for “bidirectional hierarchical predictive processing,” and thus cognize using functional “brains.” I illustrate this *via* the circadian rhythmicity of single-celled cyanobacteria (blue-green algae). The resulting view is not absurd—cognition might be continuous with life, and Bechtel (2011) has previously treated cyanobacteria under a model of cognition rooted in control theory (Bechtel, 2009). But the result should caution against employing Clark's proposal to attain some presumed goals of cognitive science.

Circadian rhythmicity (“CR”) is virtually ubiquitous among living cells. CR is the rhythmic production of some phenomenon (onset of behavior; physiological processes in brain or periphery; transcription and translation of genes) with four defining features. First, the period of the rhythm approximates 24 h (*circa* = about, *dies* = day). Second, the rhythm is *temperature-compensated*, meaning that the rhythm's period persists at ~24 h, despite fluctuating ambient temperature (unlike many textbook chemical reactions). Third, the rhythm is *entrainable*, meaning that in the presence of “*Zeitgebers*” (externally-influenced cues which indicate the current time of day), the period and phase of the rhythm will adjust to better-match environmental time-cycles. Fourth, the rhythm is *endogenously* produced, meaning that it persists with a roughly 24-h period even when *Zeitgebers* are removed. A core circadian *clock* or *pacemaker* is the hypothesized cause of such observable rhythms in a living system.

It has been suggested that the evolutionary pressures resulting in ubiquitous CR arose ~2.5 bya—the period in which the circadian clock of the blue-green alga *Synechococcus elongatus* first evolved (Edgar et al., 2012). *S. elongatus* is today a model system in CR research (for a history see Johnson and Xu, 2009; for recent overview see Mackey et al., 2011). In these unicellular cyanobacteria, the transcription and translation of virtually the entire genome is regulated by the core clock (Kondo et al., 1993; Liu et al., 1995; Johnson et al., 1996; Ito et al., 2009). The precise mechanisms of this global regulation are subject to continued investigation, and may be diverse (Nair et al., 2002; Woelfle and Johnson, 2006; Vijayan et al., 2009).

CR is intimately involved in cyanobacterial life-cycles. In some species, the clock predicts environmental light-dark cycles so as to temporally segregate two incompatible (but equally vital) metabolic processes: photosynthesis and nitrogen fixation (Johnson et al., 1996). Absent circadian regulation of these processes, photosynthesis-produced intracellular oxygen would disrupt nitrogen fixation, preventing uptake of a critical nutrient (Fay, 1992; Berman-Frank et al., 2003). In the species *S. elongatus*, it has been shown that cell-division (reproduction) is gated by the circadian clock (Dong et al., 2010). For such reasons as these, one would expect the accuracy of cyanobacterial clocks in tracking environmental light/dark cycles to affect the fitness of cells and colonies. This has been demonstrated experimentally in *S. elongatus* strain PCC 7942 (Johnson et al., 1998; Ouyang et al., 1998; Woefle et al., 2004; Woelfle and Johnson, 2009).

**Figure 1:**
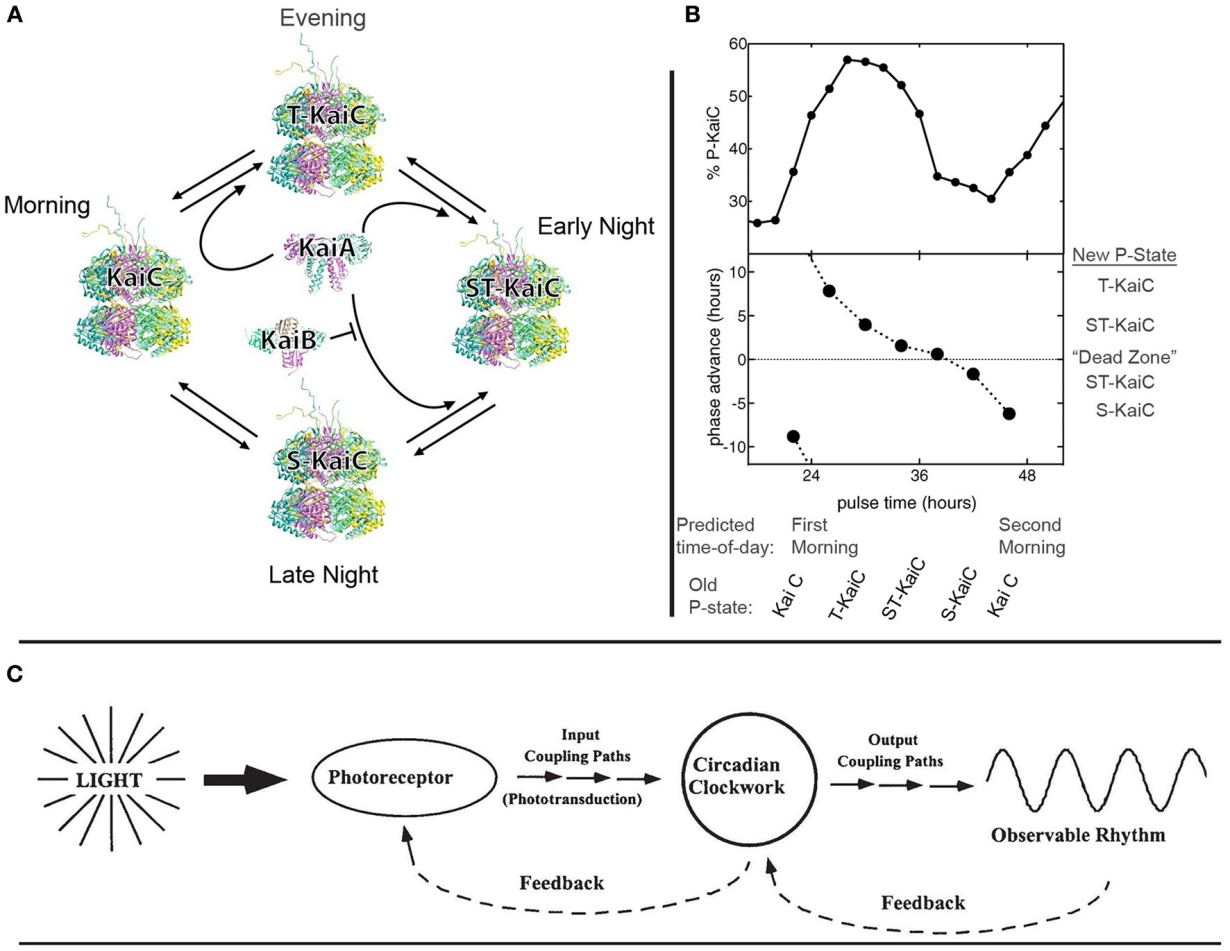
**Basic architecture and dynamics of circadian biocognition in cyanobacterium *S. elongatus* PCC 7942.** (A) The p-rhythm of KaiC constitutes the central pacemaker or “clock.” In the morning, KaiC is unphosphorylated; this p-state is here labeled as “KaiC” at left. As the day progresses toward evening, KaiA promotes the phosphorylation of KaiC at residue Threonine 432, forming p-state “T-KaiC” at top. Throughout the early night, KaiA biases T-KaiC toward further phosphorylation at residue Serine 431, forming doubly-phosphorylated “ST-KaiC,” at right. In late night, ST-KaiC begins to dephosphorylate at Threonine 432, forming “S-KaiC” at bottom. KaiB then binds to S-KaiC and inactivates the phosphorylation enhancement of KaiA, and KaiC returns to the unphosphorylated state (at left) on the next day's morning. This same cycle occurs with circadian rhythmicity in the absence of a *Zeitgeber*. [This panel modified from Dong et al. (2010) Figure 1 with permission from Elsevier]. **(B)** The endogenous p-rhythm of KaiC is directly entrained by altering the relative abundance of phosphate groups available to phosphorylate KaiC. The X-axis depicts the clock's predicted time-of-day, and the corresponding p-state of KaiC (as described in **(A)** above). The top panel depicts >1 day of circadian KaiC phosphorylation rhythms in the absence of *Zeitgebers* (first predicted morning just prior to “24 h” second predicted morning prior to “48 h”). The bottom panel shows the phase-dependent recalibration of KaiC p-rhythms, induced by providing a “pulse” of ADP, which depletes the *relative* abundance of free phosphate groups (by decreasing the ATP to ADP ratio). Recalibration occurs as an alteration of the p-state of KaiC, which encodes a new prediction regarding time-of-day. The corresponding “new” (recalibrated) p-states are depicted at right. Note the “Dead Zone,” in which no significant recalibration occurs. Outside the Dead Zone, identical pulses differentially affect the clock as a function of the currently-represented time of day, and thereby produce a variable correction of the signaled “prediction error.” The prediction error's size (how far off was the prediction?) and direction (was the clock fast or slow?), are used to either speed the clock up (positive phase advance) or slow it down (negative phase advance). [These panels modified from Rust et al. (2011). Figure 2 with permission from AAAS and the authors]. **(C)** A species-general schematic representation of the basic functional organization of a circadian system in its ecological context. Ambient light serves as the usual *Zeitgeber*, indicating the environmental time-of-day. Input pathways convey this information to the core circadian clockwork, which maintains a prediction of the current time-of-day. [In *S. elongatus*, the p-state of KaiC is fine-tuned especially by pathways involving the protein CikA (Schmitz et al., 2000; Mutsuda et al., 2003; Mackey et al., 2008)]. Output pathways to the periphery translate the clock's prediction into observable rhythms (behavior, physiological, and genetic processes, etc.) which are appropriate for the predicted time-of-day, and feedback from the periphery to the clock is used to correct prediction error. (In *S. elongatus*, see Taniguchi et al., 2010). Feedback from the clock to input mechanisms is used to modulate incoming input to the clock (see in-text discussion of photosynthesis). Any such system exhibits “bidirectional and hierarchical predictive processing,” in Clark's sense. (This panel reproduced from Lakin-Thomas and Johnson (1999) Figure 2 with permission from Elsevier).

The core pacemaker in *S. elongatus* has been identified as involving oscillations in the phosphorylation state (“p-state”) of KaiC proteins. The four stages of KaiC's phosphorylation rhythms (“p-rhythms”), and the interactions of KaiC with two regulative proteins, KaiA and KaiB, are depicted and described in Figure 1A below. As shown in Figure 1B, manipulating the relative abundance of available phosphate groups within the cell provides a direct means of biasing KaiC to a particular p-state, entraining the clock and (thereby) all downstream rhythms (Rust et al., 2011).

At any moment, the p-state of KaiC serves as a cyanobacterium's *prediction* of environmental time-of-day (Figure 1A). This prediction is used to regulate the cell's activities in a manner appropriate to the predicted time of day (Figure 1C). One important example is circadian regulation of the transcription of a gene (*PsbAI*) whose protein products are required for photosynthesis. This gene's expression is controlled by the clock so as to occur throughout predicted day, peaking prior to predicted evening (Liu et al., 1995).

In *S. elongatus*, photosynthesis is the principal means of generating ATP, making available phosphate groups for phosphorylation. Thus, acting on the prediction that daybreak is approaching (encoded in the unphosphorylated state “KaiC”) cyanobacteria initiate a process (photosynthesis) which, if the prediction were accurate, (1) would be adaptive (photosynthesis only works in sunlight), and (2) would facilitate future accuracy of the clock's predictions: successful photosynthesis produces ATP, providing the abundant phosphate groups required for progression of the p-rhythm from p-state “KaiC” (predicted morning) to “T-KaiC” (predicted dusk) and then to “ST-KaiC” (predicted early night).

More striking is the cyanobacterial response to *prediction error*. As shown in Figure 1B, the clock is differentially recalibrated to correct for prediction error, depending on whether signaled time-of-day indicates that the clock is running fast or slow. This is an instance of the feedback-loop depicted at right in Figure 1C. The signal of prediction error (relative abundance of available phosphate groups) is functionally distinguishable from the encoded prediction (p-state of KaiC) as required by Clark's proposal (Clark, in press, see esp. § 2.1). However, in an instance of the feedback-loop at left in Figure 1C, the clock continuously modulates incoming signals by regulating input processes (*psbAI* transcription and photosynthesis).

Thus, cyanobacteria actively “explain away” many incoming signals through hierarchical and bidirectional predictive processing; only “unexplained” prediction error causes recalibration. This is Clarkian cognition.

Cyanobacterial cognition involves an ancient form of forethought. The adaptiveness of accurate timekeeping may even license emotion attribution, on some construals. But on the eminently plausible assumption that unicellular algae lack phenomenal consciousness, this commentary surpasses conceivability arguments (Chalmers, 1996) and *demonstrates empirically* that applicability of Clark's model is insufficient to license attribution of consciousness. Clark's suggestive remarks in § 4 *must* be understood as applications of the same formal apparatus to a system whose phenomenology—that form of human mindedness which many cognitive scientists most wish to explain—is *presupposed on independent grounds*, not radically grounded in his account of cognition. Clark's account “accommodates” and perhaps even “illuminates” consciousness, but does not approach *explaining* it.
